# PAH101: A *GW*+BSE Dataset of 101 Polycyclic Aromatic Hydrocarbon (PAH) Molecular Crystals

**DOI:** 10.1038/s41597-025-04959-0

**Published:** 2025-04-23

**Authors:** Siyu Gao, Xingyu Liu, Yiqun Luo, Xiaopeng Wang, Kaiji Zhao, Vincent Chang, Bohdan Schatschneider, Noa Marom

**Affiliations:** 1 Department of Materials Science and Engineering, Carnegie Mellon University, Pittsburgh, PA 15213 USA; 2 Department of Physics, Carnegie Mellon University, Pittsburgh, PA 15213 USA; 3 School of Foundational Education, University of Health and Rehabilitation Sciences, Qingdao, 266113 China; 4 Department of Chemistry and Biochemistry, California State Polytechnic University at Pomona, Pomona, CA 91768 USA; 5 Department of Chemistry, Carnegie Mellon University, Pittsburgh, PA 15213 USA

**Keywords:** Electronic structure, Materials for devices, Materials for optics, Electronic materials

## Abstract

The excited-state properties of molecular crystals are important for applications in organic electronic devices. The *GW* approximation and Bethe-Salpeter equation (*GW*+BSE) is the state-of-the-art method for calculating the excited-state properties of crystalline solids with periodic boundary conditions. We present the PAH101 dataset of *GW*+BSE calculations for 101 molecular crystals of polycyclic aromatic hydrocarbons (PAHs) with up to  ~500 atoms in the unit cell. To the best of our knowledge, this is the first *GW*+BSE dataset for molecular crystals. The data records include the *GW* quasiparticle band structure, the fundamental band gap, the static dielectric constant, the first singlet exciton energy (optical gap), the first triplet exciton energy, the dielectric function, and optical absorption spectra for light polarized along the three lattice vectors. The dataset can be used to (i) discover materials with desired electronic/optical properties, (ii) identify correlations between DFT and *GW*+BSE quantities, and (iii) train machine learned models to help in materials discovery efforts.

## Background & Summary

Computational materials design and discovery requires exploring the infinitely vast chemical space using quantum mechanical methods that can reliably predict the electronic and optical properties of candidate materials. The computational cost of quantum mechanical simulations increases rapidly with the method accuracy and system size. This limits the scope of simulations that can be performed within a reasonable time in terms of the number of systems explored, their size, the accuracy of the predicted properties, and the types of phenomena that can be investigated^1–7^.

Density functional theory (DFT) is the workhorse of first-principles simulations^8^. DFT relies on approximate exchange-correlation functionals to describe the many-body quantum mechanical interactions between electrons. Computationally efficient semi-local functionals have been used extensively for high-throughput materials screening^9–18^. However, DFT is a ground-state theory, therefore it is inherently unable to describe excited-state properties of interest, such as fundamental band gaps, singlet and triplet excitation energies, optical gaps (*i.e*., the first singlet excitation energy), and optical absorption spectra. The excited states of isolated molecules may be calculated relatively efficiently with time dependent DFT (TDDFT)^19–23^. The excited states of crystalline systems may be calculated using Green’s function based many-body perturbation theory (MBPT) within the *GW* approximation and Bethe-Salpeter equation (BSE)^24–27^, which lends itself more easily than TDDFT to periodic implementations. Unfortunately, the high computational cost of *GW*+BSE simulations makes it unfeasible to use these methods for large scale materials exploration.

Machine learning (ML) may accelerate computational materials discovery by bypassing the need to perform expensive first-principles simulations^10,28–45^. To this end, statistical models are constructed based on training data to make predictions for new data points. Training ML models, especially deep neural networks (DNN), typically requires huge datasets^46,47^. Therefore, data acquisition is often the bottleneck of applying ML to computational materials discovery. With the supercomputing resources available nowadays, acquiring DFT training data with semi-local functionals is relatively fast. This has led to the proliferation of DFT datasets^28,48–55^. As a result, ML models have been trained predominantly on semi-local DFT data, which limits their applicability to structural and ground state properties. Owing to the high computational cost of *GW*+BSE, such datasets are scarce and the amount of data they contain is relatively small compared to DFT datasets^54,56,57^. We note that the *GW* datasets cited here comprise small isolated molecules, which are considerably faster to calculate than periodic molecular crystals with hundreds of atoms in the unit cell. Recently, ML has been applied to predict the *GW* quasiparticle energies of small molecules^58,59^.

It is challenging to construct transferable ML models based on “small data”. This has limited the applicability of ML to excited state properties of molecular crystals. Emerging approaches to ML with small data include multi-fidelity approaches. These methods combine a small amount of high-fidelity data with a large amount of low-fidelity data, which, although not as accurate, is sufficiently correlated with the high-fidelity data for statistical inference^60–69^. Recently, high-quality results have been achieved by fine-tuning a pre-trained DNN model with small datasets or combining feature selection with DNN^70,71^. Other approaches involve using low-fidelity features, selected based on physical/chemical knowledge, to construct surrogate models that are predictive of high-fidelity data. One such approach is the sure-independence-screening-and-sparsifying-operator (SISSO)^72,73^ ML algorithm. The input of SISSO is a set of primary features, which are physical descriptors that could be correlated with the target property. SISSO generates a huge feature space by iteratively combining the primary features using linear and nonlinear algebraic operations. Subsequently, linear regression is performed to identify the most predictive models. Physical and chemical knowledge is leveraged in the choice of primary features and in the rules for combining them. SISSO has been demonstrated to work well with a relatively small amount of data for several different types of materials systems and properties^13,74–92^.

One application that requires predicting the excited-state properties of molecular crystals is singlet fission (SF), the conversion of one singlet exciton into two triplet excitons^93–96^. The efficiency of solar cells can be boosted by augmenting traditional absorbers with SF materials^97–99^. The SF material can convert photons with energies high above the traditional absorber’s band gap into two charge carriers instead of losing their excess energy to heat. Currently, few classes of materials are known to undergo intermolecular SF in the solid state, and insufficient stability under operating conditions precludes their utilization in commercial modules^93,94,100,101^. Therefore, there is a need for computational discovery of new SF materials. The primary criterion for a material to undergo SF is the thermodynamic driving force, *i.e*., the difference between the singlet exciton energy and twice the triplet exciton energy, *E*_S_ − 2*E*_T_, which can be calculated using *GW*+BSE^102–106^.

**Fig. 1:**
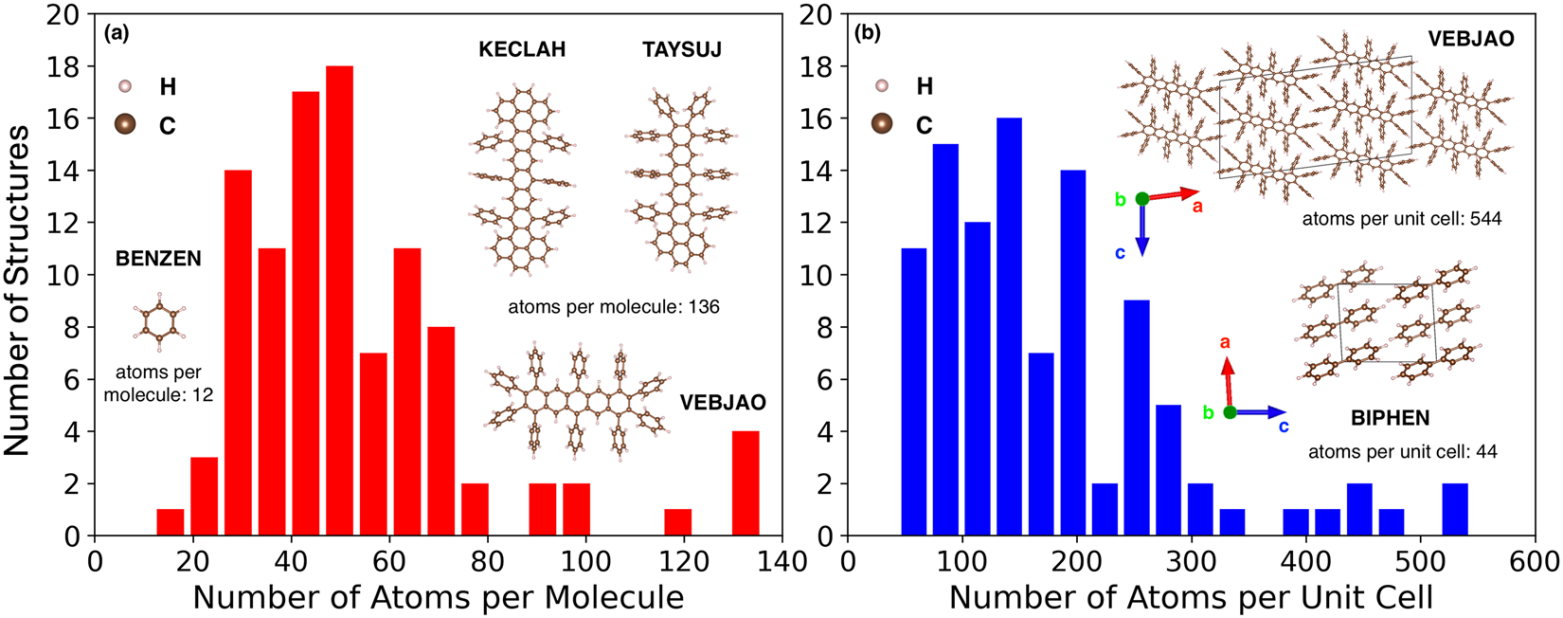
Statistics of PAH101. Histograms of the number of atoms (**a**) in a single molecule and (**b**) in a crystal unit cell for the materials in the PAH101 set. Also shown are illustrations of the molecular structures of benzene (BENZEN), 9,11,13,22,24,26-Hexaphenyltetrabenzo[*de*, *rs*, *wx*, *k*_1_*l*_1_]nonacene (KECLAH), 9,11,13,14,15,16,18,20-Octaphenyldibenzo[*de*, *c*_1_*d*_1_]heptacene (TAYSUJ), 1,2,3,4,6,8,9,10,11,13-Decaphenylpentacene (VEBJAO), and the crystal structures of 1,2,3,4,6,8,9,10,11,13-Decaphenylpentacene (VEBJAO) and Biphenyl (BIPHEN).

Recently, we have used SISSO to find models based on low-cost DFT properties that can reliably predict the *GW*+BSE SF driving force^107^. SISSO generated several models that predicted the *GW*+BSE SF driving force with errors below 0.2 eV. Based on considerations of accuracy and computational cost, two SISSO models were selected to build a two-step hierarchical classifier for screening promising candidates for SF. To train SISSO, we generated a dataset of *GW*+BSE calculations of the SF driving force of 101 molecular crystals of polycyclic aromatic hydrocarbons (PAHs). PAHs are compounds comprising carbon and hydrogen atoms and containing multiple aromatic rings. Most SF materials are PAHs. In addition to SF, PAHs and their functionalized derivatives have versatile applications in organic electronic devices^108–118^. To form the PAH101 set, crystal structures of unsubstituted PAHs (containing only C and H atoms) were extracted from the Cambridge Structural Database (CSD)^119^. The PAH101 set contains several sub-classes including acenes, rylenes, zethrenes, as well as various compounds that do not belong to any particular family. As shown in Fig. 1, the PAH101 set contains molecules ranging in size from 12 atoms in benzene (CSD Reference: BENZEN) to 136 atoms in two pyrene-stabilized acenes 9,11,13,22,24,26-Hexaphenyltetrabenzo[*de*, *rs*, *wx*, *k*_1_*l*_1_]nonacene (CSD Reference: KECLAH), 9,11,13,14,15,16,18,20-Octaphenyldibenzo[*de*, *c*_1_*d*_1_]heptacene (CSD Reference: TAYSUJ), and a phenylated pentacene 1,2,3,4,6,8,9,10,11,13-Decaphenylpentacene (CSD Reference: VEBJAO). The crystal size in the PAH101 set ranges from 44 atoms in the unit cell for biphenyl (CSD Reference: BIPHEN) to 544 atoms in 1,2,3,4,6,8,9,10,11,13-Decaphenylpentacene (CSD Reference: VEBJAO).

The PAH101 dataset contains *GW*+BSE results for the electronic and optical properties of molecular crystals, as well as the DFT-level SISSO primary features used in Ref. ^107^. We envision this dataset being used for computational discovery of crystalline organic semiconductors and chromophores with desired properties for applications in various organic electronic devices. The electronic and optical properties of most of the materials in the PAH101 set have not been thoroughly investigated experimentally. Some of the quantities calculated here, such as triplet excitation energies, are difficult to probe experimentally and require highly specialized techniques and facilities. Therefore, although the PAH101 set is relatively small, it is possible that some useful materials would be found in it. For example, the dataset contains information on optical gaps and absorption spectra, which could be used to search for chromophores that absorb light in a certain energy range. In addition, the dataset contains singlet and triplet excitation energies, which can be used to evaluate candidate chromophores for triplet-triplet annihilation (TTA) and thermally activated delayed fluorescence (TADF). TTA chromophores can be used for harvesting photons with energies below the absorption threshold of a solar cell by up-conversion of two low-energy triplet excitons into one singlet exciton that can be absorbed^120,121^. TADF chromophores can be used to enhance the efficiency of OLEDs by converting electrically generated non-radiative triplet excitons into radiative singlet excitons^23,122,123^.

The dataset also contains quantities related to charge separation and transport in organic devices. The band dispersion, which can be extracted from *GW* band structures in the dataset, is related to transport in crystalline organic semiconductors, which affects the performance of organic electronic devices such as field effect transistors (OFETs)^124^. The singlet exciton binding energy corresponds to the difference between the *GW* fundamental gap and the BSE optical gap. This is the energy required to split photogenerated excitons into free charge carriers in organic solar cells. In most organic materials the exciton binding energy is significant compared to inorganic materials because the dielectric screening of charges is not as strong. However, some materials in the PAH101 set, characterized by very extended and/or elongated *π* systems, have low exciton binding energies, below 0.2 eV. The *GW* static dielectric constant is related to the strength of charge screening in the material, and consequently to the exciton binding energy and band dispersion. Organic materials typically have relatively low dielectric constants (around 3). The PAH101 set contains several materials with unusually high dielectric constants, ranging from 7 to 10.

Furthermore, this dataset can be used as a resource for comparing and benchmarking the performance of various electronic structure methods for calculating the electronic and optical properties of molecular crystals. Finally, this dataset can be used to augment other datasets, e.g., DFT datasets for molecular crystals or TDDFT datasets for isolated molecules to train multi-fidelity ML models for predicting various electronic and optical properties of molecular crystals. In summary, because the PAH101 is a unique set of *GW*+BSE data for molecular crystals, we expect it to be a resource of great usefulness to the computational community.

## Methods

### Hydrogen Addition

**Fig. 2:**
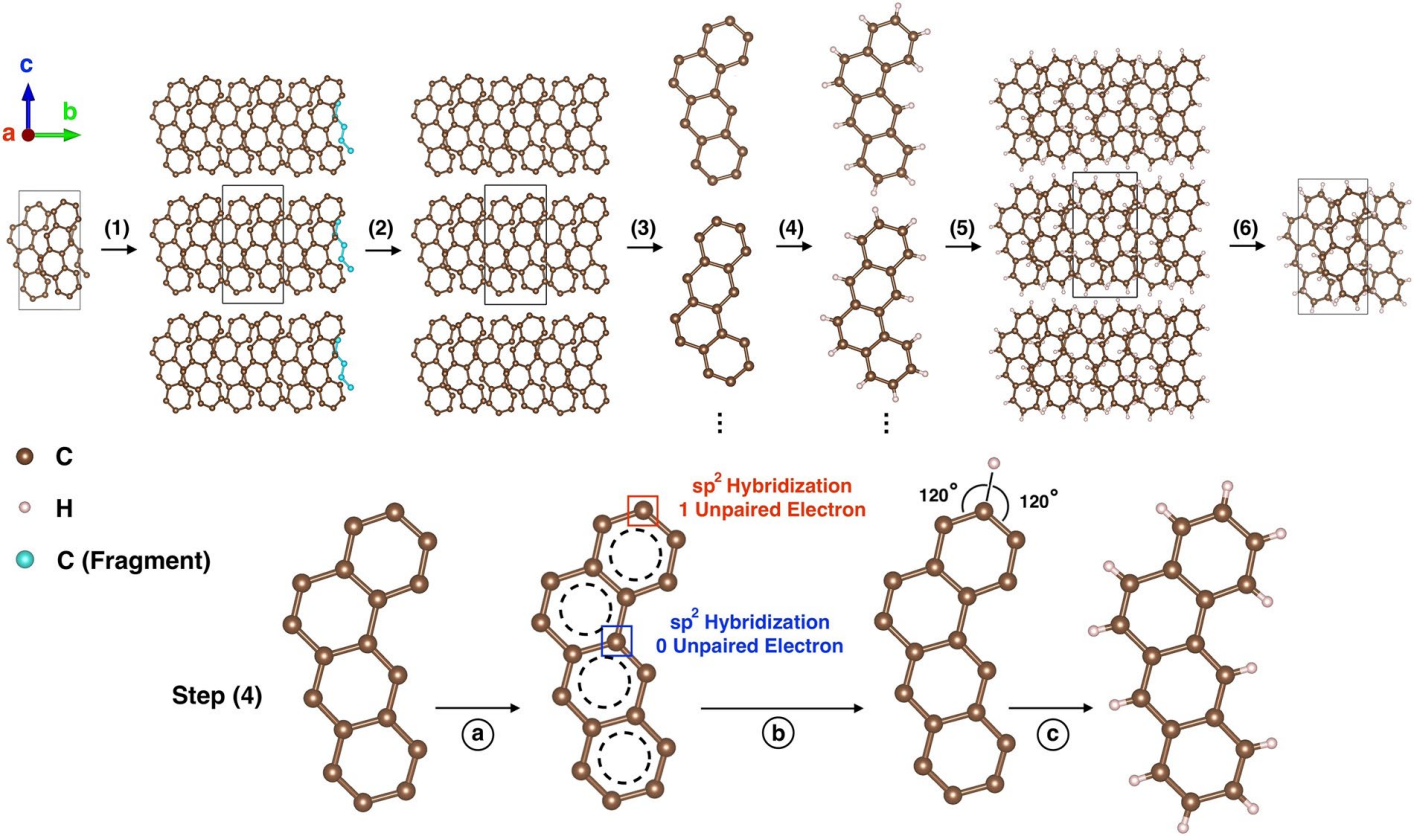
Hydrogen addition. Schematic illustration of the workflow of adding missing H atoms with HAppend, demonstrated for the BEANTR crystal. The top row shows the steps of (1) super-cell construction, (2) removal of broken molecular fragments (colored in blue), (3) extraction of molecules, (4) addition of H atoms to all molecules, (5) reconstruction of the supercell with the H atoms attached to all molecules, and (6) reduction of the supercell to a single unit cell. For Steps (3) and (4) only two molecules are shown for clarity. The bottom row presents a detailed view of the hydrogen addition step: (**a**) identification of missing H sites, (**b**) calculation of approximate H atom positions, and (**c**) attachment of H atoms to the molecule.

The starting geometries of the 101 molecular crystals were extracted from the Cambridge Structural Database (CSD)^119^. The CSD reference codes for each material are available in the data records. Some of the CIF files in the CSD are missing the hydrogen atom positions, which cannot be determined by X-ray diffraction. To provide an approximate position for each missing H atom, we have developed the Hydrogen Append (HAppend) code, available in the GitHub repo: https://github.com/BLABABA/HAppend. HAppend is written in Python and uses RDKit^125^ and Pymatgen^126^. The workflow of HAppend is illustrated in Fig. 2 using BEANTR as an example. All H atoms were removed from the CIF file for the purpose of demonstration. HAppend does not use the symmetry information provided in the CIF file. In step (1) the unit cell is replicated to build a super-cell so that any molecular fragments inside the unit cell can be completed. In step (2) all the complete molecules and molecular fragments are identified. Subsequently, any broken fragments at the supercell boundary (colored in blue in Fig. 2) are removed. In step (3) all the complete molecules are extracted. Only two molecules are shown in Fig. 2 for demonstration purposes. Step (4) is identifying the missing hydrogen sites and appending H atoms to each molecule. A detailed schematic of step (4) is shown in the bottom row of Fig. 2. In step 4a the missing hydrogen sites are identified by checking the type of hybridization of each carbon atom against the number of valence electrons participating in covalent bonds. In this example, all C atoms in the aromatic rings have sp^2^ hybridization. In step 4b H atoms are attached to atoms with unpaired valence electrons. The bond length and angle are determined based on the bonded neighbors and hybridization type. In this example, given that the C atom is sp^2^ hybridized, the two H-C-C angles should be about 120°. This process is performed for all atoms in the BEANTR molecule and the completed molecule is obtained after step 4c. Step (5) reconstructs the complete super-cell with appended H atoms. Step (6) reduces the super-cell back to the original unit cell with all the coordinates for the missing H atoms now known. Finally, sanity checks are performed to verify that the structure is correct. The structure is checked against the expected chemical formula (if provided in the CIF file from the CSD). In addition, RDKit is used to repeat step 4b and confirm that the explicit valence matches with the type of hybridization for each atom. If the sanity check fails, the user may have to attach H atoms manually. HAppend is not limited to PAHs and may be used to add missing H atoms to other types of organic molecules.

### Structural Relaxation

Full unit cell relaxation was performed with either CASTEP^127^ or FHI-aims^128,129^ (which code was used is reported in the data records). The Perdew, Burke, and Ernzerhof (PBE) exchange-correlation functional^130^ was used with the Tkatchenko-Scheffler (TS) pairwise dispersion method^131^. For relaxations performed with CASTEP, norm-conserving pseudopotentials were utilized for carbon and hydrogen. The plane-wave basis set cutoff was 750 eV. A Monkhorst-Pack k-grid with a spacing of 0.07 Å^−1^ was adopted. The convergence thresholds for total energy, maximum force, maximum stress, and maximum displacement were 5 × 10^−6^ eV/atom, 0.01 eV/Å, 0.02 GPa, and 5 × 10^−4^ Å^−1^, respectively. For structures relaxed with FHI-aims, the tight numerical settings and tier-2 basis sets were used. The fully relaxed crystal structures and the molecular geometries extracted from them are provided in the data records. The *GW*+BSE calculations were performed for the fully relaxed crystal structures.

### DFT Features

The data records include the DFT primary features used for SISSO in Ref. ^107^. The DFT features of molecules and crystals were calculated using FHI-aims^128,129^. From considerations of computational efficiency, the DFT primary features were calculated with locally-optimized geometries. The crystal structures were relaxed with the lattice vectors fixed at the experimental values and the single molecule properties were calculated using molecules extracted from these locally-optimized crystal structures. All primary features were calculated with the PBE functional^130^. using the tight numerical setting and tier-2 basis sets of FHI-aims^128^. This procedure, followed in Ref. ^107^, was intended to simulate a screening workflow, in which the primary features are evaluated fast and further more accurate calculations are pursued only for materials predicted to be promising by the SISSO models.

### Mean-Field Wave-Function Calculation

The Quantum ESPRESSO package^132^ was used to compute the DFT eigenvectors and eigenvalues, which served as the starting point for non-self-consistent *GW*+BSE calculations, using the PBE functional. The kinetic energy cutoff was 50 Ry. The k-point grids used for each material are reported in the data records. Norm-conserving pseudopotentials were chosen in order to take advantage of the simplification of matrix elements in *GW*+BSE calculations^133^. We used the Troullier-Martins norm-conserving pseudopotentials provided on the Quantum Espresso website. These were generated using FHI98PP^134^ and converted with fhi2upf.x v.5.0.2 to the Quantum Espresso format. The cutoff radii used for carbon (C) were 1.5 Bohr for *s* states and 1.5 Bohr for *p* states. For the *s* states of hydrogen (H) a cutoff radius of 0.8 Bohr was used. The reference configurations for C and H were 2*s*^2^2*p*^2^ and 1*s*^1^, respectively. The pseudopotential files used in our calculations are provided in the GitHub repository HAppend/UPF/.

### *GW*+BSE Calculations

The BerkeleyGW package^133^ was used to perform *GW*+BSE calculations. From considerations of computational cost, non-self-consistent *G*_0_*W*_0_ calculations were performed. 550 unoccupied states were included in the dielectric function and the self-energy operator evaluations. The inverse microscopic dielectric function was calculated in the static limit at zero frequency. The generalized plasmon-pole model was used to obtain the self-energy at finite frequency. The static remainder correction^135^ was applied to accelerate convergence. The screened Coulomb cutoff was set to 10 Ry. For all other settings the default values of BerkeleyGW were used. The Bethe-Salpeter equation was solved within the Tamm-Dancoff approximation (TDA) with 24 valence bands and 24 conduction bands included. The fine k-point grid wave-functions were generated using a fine k-point grid twice as dense as the coarse k-point grid. The coarse and fine k-point grid settings for each material are reported in the data records.

## Data Records

The PAH101 dataset^136^ is available via the NOvel MAterials Discovery (NOMAD) repository^137^ and can be accessed at 10.17172/NOMAD/2024.12.05-1. The data are provided in YAML (.yaml) format. Each file is named as *CSD-REFERENCE*.archive.yaml, where *CSD-REFERENCE* is the CSD reference code for each structure. The data structure for each material record is described in Table 1. The top level sections are *struct_id*, *geometry*, *dft*, and *gwbse*. The *struct_id* section contains the CSD reference code. The *geometry* section provides the fully relaxed crystal structure and the single molecule geometry extracted from it. The *dft* section contains all the SISSO primary features used in Ref. ^107^. The *gwbse* section provides quasi-particle (QP) and excitonic properties for the PAH101 crystals, including the fundamental gap, quasiparticle band structure, the static dielectric constant, the first singlet exciton energy (optical gap), the first triplet exciton energy, the full dielectric function, and optical absorption spectra for light polarized along the three lattice vectors. The *GW* static dielectric constant is not available for some of the materials in the dataset because some data that was not needed for Ref. ^107^ was not preserved.

**Table 1:** Data records: Description of the data structure of the PAH101 set with explanations of all entries. Abbreviated notations for the DFT primary features are provided in parentheses.

*struct*_*id*	the CSD reference code for this structure		
*geometry*	*relaxed*_*crystal*	the DFT-relaxed crystal structure saved in Pymatgen Structure format	
	*molecule*	the single molecule geometry extracted from *relaxed*_*crystal*, saved in Pymatgen Molecule format	
	*chemical*_*formula*	chemical formula of the single molecule	
	*relax*_*code*	code used to perform crystal structure relaxation	
*dft*	*gap*_*s* (Gap^S^)	the single molecule gap, calculated based on the energy difference between the highest occupied molecular orbital (HOMO) and the lowest unoccupied molecular orbital (LUMO)	
	*Et*_*s* (${E}_{{\rm{T}}}^{{\rm{S}}}$)	the single molecule triplet formation energy, calculated based on the total energy difference between the ground-state and triplet-state molecule	
	*DF*_*s*	the single molecule DFT estimate for the SF driving force, calculated by taking the difference between *gap*_*s* and twice *Et*_*s*	
	*IP*_*s* (IP^S^)	the single molecule ionization potential (IP), calculated based on the total energy difference between a cation and neutral molecule	
	*EA*_*s* (EA^S^)	the single molecule electron affinity (EA), calculated based on the total energy difference between an anion and neutral molecule	
	*bandgap* (Gap^C^)	the crystal band gap	
	*Et* (${E}_{{\rm{T}}}^{{\rm{C}}}$)	the crystal triplet formation energy, calculated based on the total energy difference between the ground-state and triplet-state crystal	
	*DF*	the crystal DFT estimate for the SF driving force, calculated by taking the difference between *bandgap* and twice *Et*	
	*VBdisp* (${{\rm{VB}}}_{{\rm{disp}}}^{{\rm{C}}}$)	the valence band dispersion, *i.e*., the energy range of the HOMO-derived band	
	*CBdisp* (${{\rm{CB}}}_{{\rm{disp}}}^{{\rm{C}}}$)	the conduction band dispersion, *i.e*., the energy range of the LUMO-derived band	
	*h*_*ab*_ (*H*_ab_)	the transfer integral, calculated with fragment orbital DFT^172^	
	*polarization* (PolarTensor^S^)	the trace of the polarization tensor for a single molecule, calculated with DFT using the PBE functional and the range-separated self-consistently screened version of many-body dispersion (MBD@rsSCS) method^143,173^	
	*epsilon*_*mbd* (*ϵ*^C^)	the dielectric constant calculated with PBE+MBD@rsSCS	
	*weight*_*s* (MolWt^S^)	the molecular weight in atomic mass units (amu)	
	*density* (*ρ*^C^)	the crystal density in amu Å^−3^	
	*eigenvalues*	the eigenvalues for the single molecules, data stored as *n* × 4 matrix, whose columns are: State, Occupation, Eigenvalue [Ha], Eigenvalue [eV]	
	*kgrid*	the *k*-grid settings for the calculation of crystal primary features	
*gwbse*	*absorption*	*a*	Optical absorption spectrum for light polarized along the a, b, and c crystal axes. Each absorption data record contains four columns: energy (eV), the imaginary and real parts of the dielectric function *ϵ*_2_ and *ϵ*_1_, and the normalized joint density of states.
		*b*	
		*c*	
	*bandstructure*	*kpoints*	the high-symmetry *k*-point path used to calculate the *GW* band structure
		*val*	the values of band structure, saved as *n* × 8 matrix, whose columns are: spin, band index, k-point coordinate x, k-point coordinate y, k-point coordinate z, mean-field energy, quasi-particle energy, difference between mean-field and quasi-particle energy
	*bse*_*Es*	the singlet exciton energy (optical gap) calculated with BSE	
	*bse*_*Et*	the triplet exciton energy calculated with BSE	
	*bse*_*DF*	the SF driving force for a crystal, *bse*_*Es* − 2 × *bse*_*Et*	
	*kgrid*_*coarse*	the *k*-grid used for coarse grid wave-function calculation	
	*kgrid*_*fine*	the *k*-grid used for fine grid wave-function calculation	
	*fundamental*_*gap*	the fundamental gap calculated with *GW*	
	*bse*_*Es*_*bind*	the singlet-state exciton binding energy	
	*bse*_*Et*_*bind*	the triplet-state exciton binding energy	
	*epsilon*_*gw*	the static dielectric constant calculated within the random phase approximation (RPA); N.A. entered if not available	

## Technical Validation

### Crystal Structures

**Fig. 3:**
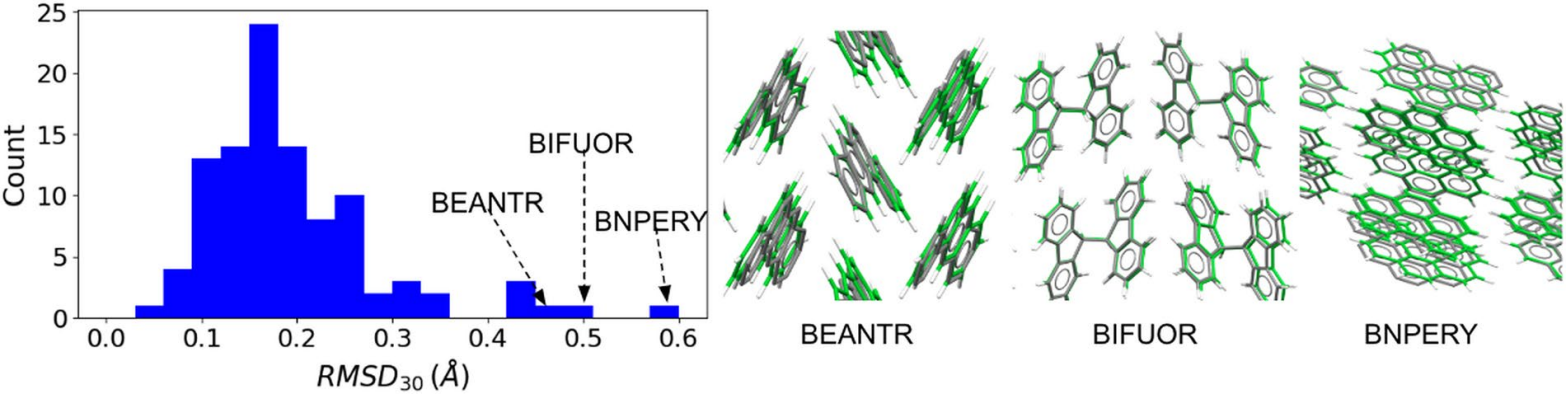
Crystal structure validation. Histogram of the *RMSD*_30_ of crystal structures relaxed with PBE+TS compared to the experimental structures from the CSD for the PAH101 set. The similarity overlay plots generated by Mercury are shown for BNPERY, BIFUOR, and BEANTR with the experimental structures colored in gray and the relaxed structures colored in green.

To verify the results of full unit cell relaxation with PBE+TS, the root-mean square distance (RMSD) between the relaxed structures and the experimental structures was calculated. We used the COMPACK^138^ molecular overlay method, implemented as the Crystal Packing Similarity tool, in Mercury 2023.2.0^139^. COMPACK overlays clusters of molecules taken from each crystal, within given distance and angle tolerances, and minimizes the RMSD between atoms, typically excluding hydrogen. The output of COMPACK is the number of molecules that could be overlaid and the RMSD. COMPACK comparisons were performed with a cluster of 30 molecules and distance and angle tolerances of 35% and 35°. H atoms were not included. These were the settings used for structure comparison in the 7^*th*^ crystal structure prediction blind test^140,141^. Figure 3 shows a histogram of the RMSD obtained for the PAH101 set. For the majority of the structures in the dataset the RMSD is below 0.3 Å. The three structures with the largest RMSDs are BNPERY, BIFUOR, and BEANTR. All three are monoclinic structures with larger than average deviations in their *b* lattice parameter and *β* angle. For instance, the relaxed *b* parameter of BEANTR is 6.00 Å, compared to 6.50 Å in the experimental structure, a deviation of 7.7 %. The relaxed structure of BNPERY has a *β* angle of 92.2°, compared to 98.5° in the experimental structure. Some differences between structures relaxed by DFT at 0K and structures experimentally characterized at room temperature are to be expected^142^. Overall, the performance of PBE+TS is within the community accepted standards of agreement with experiment, as established in the crystal structure prediction blind tests^140,141^. It is possible that performing relaxations with the more accurate many-body dispersion (MBD)^143^ method would reduce the RMSD.

### *GW*+BSE Convergence

**Fig. 4:**
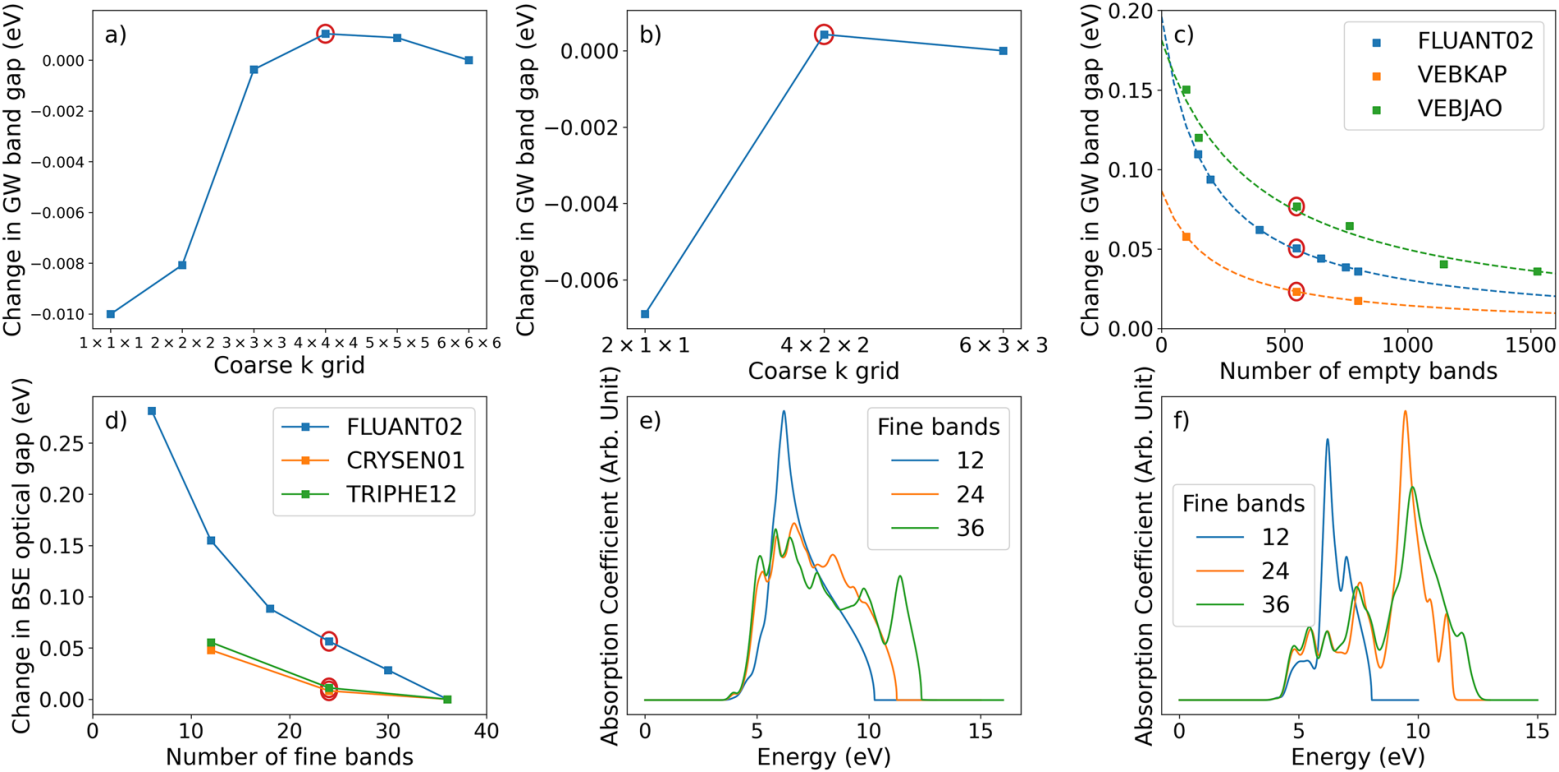
Convergence of *GW*+BSE calculations. Change in the *GW* band gap as a function of the coarse *k*-point grid for (**a**) benzene and (**b**) 9,9’-bifluorenyl (BIFUOR) with respect to the finest *k* grid considered. (**c**) Change in the *GW* band gap as a function of the number of empty bands for fluoranthene (FLUANT02), 6-phenylpentacene (VEBKAP), and 1,2,3,4,6,8,9,10,11,13-decaphenylpentacene (VEBJAO) with respect to the extrapolated value. The dashed lines are a hyperbolic fit to the data. (**d**) Change in the optical gap of fluoranthene, chrysene (CRYSEN01), and triphenylene (TRIPHE12) as a function of the number of valence and conduction bands used in the BSE step with respect to the highest number of bands considered. The chosen settings are circled in red in Panels a-d. Absorption spectra obtained using an increasing number of bands in the BSE step for light polarized along the *a*-axis of (**e**) chrysene and (**f**) triphenylene.

The results of *GW*+BSE calculations with the BerkeleyGW code are sensitive to the convergence of several parameters^57,144,145^. Because of the large number of calculations performed for the PAH101 set, we have chosen parameters that provide a balance between accuracy and computational cost. The convergence of the settings used for the PAH101 dataset have been demonstrated previously to be sufficient for selected systems^102,107^. Figure 4a,b shows the convergence with respect to coarse k-point grid used in the *GW* step for representative materials. The number of k-points is inversely proportional to the unit cell size. Benzene has the smallest unit cell in the PAH101 set and therefore requires a relatively large number of k-points. 9,9’-bifluorenyl (CSD reference code BIFUOR) represents a system of intermediate size. For both materials, increasing the number of k-points beyond the chosen settings leads to a change of less than 0.001 eV in the *GW* band gap.

Figure 4c shows the convergence with respect to the number of bands used in the *GW* step for the representative materials fluoranthene (CSD reference code FLUANT02), 6-phenylpentacene (CSD reference code VEBKAP), and 1,2,3,4,6,8,9,10,11,13-decaphenylpentacene (CSD reference code VEBJAO). The latter has the largest number of atoms in the unit cell in the PAH101 set and is therefore expected to require the most empty bands. To extrapolate the *GW* band gap to the limit of an infinite number of empty bands we applied a hyperbolic fit^146^: *f*(*N*) = *a*/(*N* − *N*_0_) + *b*, where *N* is the number of bands, *a*, *b*, and *N*_0_ are fitting parameters. For all three materials the difference between the *GW* band gap obtained with 550 empty bands and the extrapolated value is below 0.08 eV.

Figure 4d shows the convergence with respect to the number of fine bands used in the BSE step for the representative examples of fluoranthene, triphenylene (TRIPHE12), and chrysene (CRYSEN01). Increasing the number of valence and conduction bands used for the BSE step beyond 24 leads to a change of less than 0.06 eV in the optical gap. Figure 4e,f shows the absorption spectra obtained with an increasing number of fine bands for light polarized along the *a*-axis of chrysene and triphenylene. In both cases, the spectrum obtained with 12 fine bands is clearly unconverged. The spectra obtained with 24 and 36 fine bands are similar up to about 8 eV for chrysene and about 9 eV for triphenylene. In general, if one wishes to converge the absorption spectrum tightly up to a high energy, then a larger number of fine bands should be used.

The settings used for the PAH101 set are sufficiently robust for “production” calculations. Notably, in the time that passed since the PAH101 set was generated, there have been advances in streamlining the convergence of MBPT calculations^147–150^. These have focused primarily on inorganic crystals with a few atoms in the unit cell. A workflow that converges the settings for each system individually would be too expensive for systems of the size of the PAH101 set. If a certain material is of particular interest, then more detailed calculations may be pursued with ultra-converged settings and/or more accurate methods than *G*_0_*W*_0_@PBE.

### Optical Absorption

The *GW*+BSE approach has been benchmarked extensively for isolated molecules, for which high-level quantum chemistry reference data can be calculated^57,151–155^. For molecular crystals no benchmark studies are available, owing to the difficulty of obtaining reference data for large systems with periodic boundary conditions. Therefore, we are only able to validate the results of *GW*+BSE by comparison to experiments. Table 2 shows a comparison of the *GW*+BSE optical gaps (singlet exciton energies) to experimental values and *GW*+BSE values reported by others, where available. The *GW*+BSE values reported here are within 0.2 eV or less of the values reported by others. The results of *GW*+BSE calculations can differ because of differences in the implementation and convergence settings, as discussed extensively in Ref. ^57^. Because the absorption edge is not abrupt, Tauc plots are typically used to extract the optical gap from absorption spectra^156–159^. This can lead to some uncertainty in the experimental values. Here, if multiple experimental values are found for the same material, they are within 0.1 eV or less of each other in most cases. For the entries marked with *, we used the Tauc method to extract the optical gap from the experimental data because no value for the optical gap was reported in the paper. For the entries marked with **, there is a larger uncertainty in the optical gaps extracted using the Tauc method because the absorption edge does not decay to zero. In most cases, the *GW*+BSE optical gaps are within 0.2 eV or less from experimental values.

**Table 2:** Optical gaps obtained using *GW*+BSE (${E}_{g}^{GW+BSE}$) compared with experimental values (${E}_{g}^{Exp}$) and *GW*+BSE values reported by others (${E}_{g}^{GW+BSE}$ in literature), where available.

CSD Ref. Code	Compound Name	${{\boldsymbol{E}}}_{{\boldsymbol{g}}}^{{\boldsymbol{G}}{\boldsymbol{W}}+{\boldsymbol{B}}{\boldsymbol{S}}{\boldsymbol{E}}}$ (eV) in PAH101	${{\boldsymbol{E}}}_{{\boldsymbol{g}}}^{{\boldsymbol{E}}{\boldsymbol{x}}{\boldsymbol{p}}}$ (eV)	${{\boldsymbol{E}}}_{{\boldsymbol{g}}}^{{\boldsymbol{G}}{\boldsymbol{W}}+{\boldsymbol{B}}{\boldsymbol{S}}{\boldsymbol{E}}}({\boldsymbol{e}}{\boldsymbol{V}})$ in literature
BENZEN	Benzene	4.83	4.69-4.8^174^	5.0^167^
ANTCEN	Anthracene	3.22	3.16^175^	3.3^167^
TETCEN01	Tetracene	2.24	2.38^176,177^	2.4^167^
PENCEN	Pentacene	1.72	1.8-1.85^178–180^	1.7-1.8^167,181,182^
ZZZDKE01	Hexacene	1.17	1.37*-1.4^183–185^	1.0^167^
QQQCIG04	Rubrene (Orthorhombic)	2.28	2.32^186^	
QQQCIG13	Rubrene (Monoclinic)	2.62	2.36^187^	
QQQCIG14	Rubrene (Triclinic)	2.30	2.31^187^	
PERLEN05	Perylene (SHB)	2.61	2.58*^188,189^	
PERLEN07	Perylene (HB)	2.45	2.49*^188,189^	
POBPIG	Diindeno[1,2,3-cd:1′,2′,3′-lm]perylene	2.21	2.25^190^	
QUATER10	Quaterrylene	1.33	1.48-1.60^134,191,192^	
CORONE01	Coronene	2.96	2.9-2.92*^193,194^	
HBZCOR	Hexabenzo(bc,ef,hi,kl,no,qr)coronene	2.70	2.80^142,162^	
BEANTR	1,2-Benzanthracene	3.27	3.14^195^	
BIPHEN	Biphenyl	3.41	4.1-4.18^196–199^	
CRYSEN01	Chrysene	3.66	3.6**^160^	
TERPHE02	p-Terphenyl	4.17	3.9**^160^	
BNPERY	1,12-Benzoperylene	2.80	2.4-2.5*^200^	
KUBVUY	10,10’-Diphenyl-9,9’-bianthryl	3.23	2.9*^201^	
KUBWAF01	9,9’-Bianthracenyl	3.05	2.7-2.8*^202^	

Entries marked with * were extracted by us from absorption spectra using the Tauc method. Entries marked with ** have an absorption spectrum that is non-zero in the low-energy region, leading to a larger uncertainty in the optical gaps extracted using the Tauc method.

**Fig. 5:**
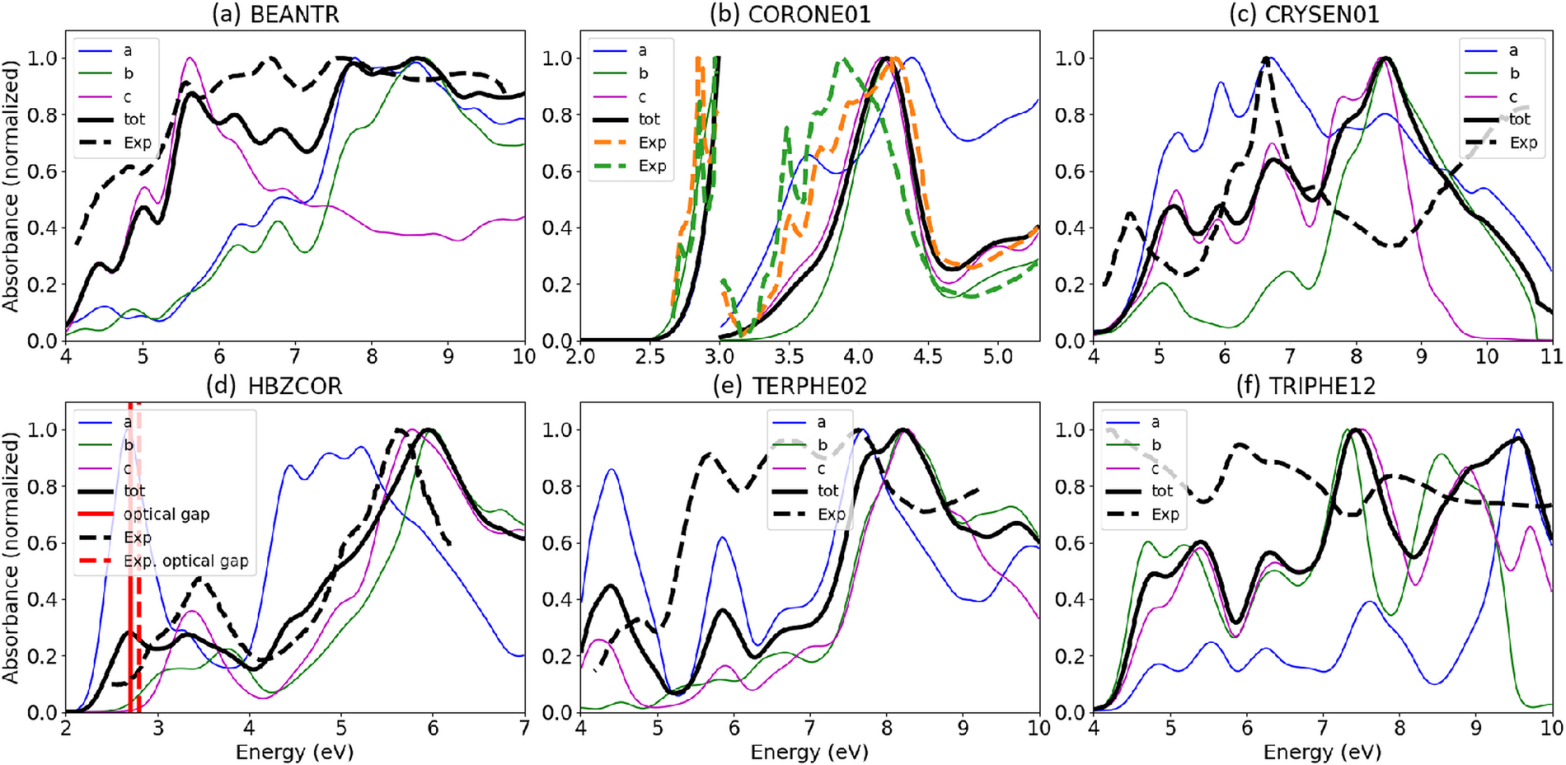
Absorption spectra. Absorption spectra calculated using *GW*+BSE compared with thin film experiments^160–162^ for (**a**) 1,2-benzanthracene (BEANTR), (**b**) coronene (CORONE01) with the region around the absorption edge magnified for clarity, (**c**) chrysene (CRYSEN01), (**d**) hexabenzo(bc,ef,hi,kl,no,qr)coronene (HBZCOR), (**e**) p-terphenyl (TERPHE02), and (**f**) triphenylene (TRIPHE12).

The *GW*+BSE absorption spectra are validated by comparison to thin film experimental data for representative materials^160–162^. For an anisotropic crystal, the absorbance depends on the polarization direction of the incident light. Most absorption experiments are performed on polycrystalline samples and even in experiments performed on single crystals the crystallographic orientation of the sample with respect to the polarization of the incident light is often unknown. This introduces some uncertainties in the comparison with experiments. We calculate the absorbance for light polarized along the *a*, *b*, and *c* lattice vectors and normalize the maximum of the total absorbance to one. The results are shown in Fig. 5. For 1,2-benzanthracene (BEANTR), coronene (CORONE01), and hexabenzo(bc,ef,hi,kl,no,qr)coronene (HBZCOR) the agreement of the *GW*+BSE spectra with experiment is very good. For chrysene (CRYSEN01), p-terphenyl (TERPHE02), and triphenylene (TRIPHE12) the agreement is more qualitative.

In addition to the unknown direction of the polarization with respect to the crystal axes, there are other factors, both on the experimental side and on the theoretical side that can contribute to discrepancies. In ref. ^160^ the crystal structure of the films is not reported. The crystal structures used in our calculations are the common forms of p-terphenyl and chrysene, but both materials have other polymorphs reported in the CSD (for triphenylene all CSD entries appear to be the same structure but we cannot rule out the appearance of a different thin film polymorph). In polycrystals there can be contributions from grain boundaries (in samples comprising very small crystallites, which is not the case here, there can be surface contributions as well). Furthermore, we do not consider vibrational contributions in our simulations. Sources of errors in *GW*+BSE calculations include the DFT exchange-correlation functional used for the mean-field starting point, numerical convergence of various settings (k-point grids, number of empty states used in the *GW* step, the number of bands used in the BSE step), the non-self-consistency in the *GW* step, the plasmon pole approximation used in the *GW* step^57^, the Tamm-Dancoff approximation used in the BSE step^25,163–168^, and the static approximation for *W* used in the BSE step^163,169^. See also Ref. ^170^ for additional discussion. The significance of different sources of errors can be material dependent. In the future, it would be desirable to rigorously assess the contributions of different sources of errors in *GW*+BSE by comparison to high-level theories or well-controlled experiments (performed on single crystals with well-defined polarization) for a diverse benchmark set of molecular crystals.

## Usage Notes

The PAH101 set is the currently the largest trove available of *GW*+BSE data for molecular crystals. As such, it offers unique opportunities to (i) discover materials with desired electronic/ optical properties in the dataset itself, (ii) learn about correlations between DFT and *GW*+BSE values of various properties, and (iii) train machine learning models to help in materials discovery efforts. Examples of these use cases are provided in the Supplementary Information (SI).

The prospect of discovery of materials with potentially useful electronic and optical properties for organic devices is demonstrated in the SI. The PAH101 dataset contains materials with a broad range of optical gaps and triplet exciton energies. Based on the singlet and triplet excitation energies materials can be evaluated as prospective candidates for SF, TTA, and TADF to improve the efficiency of organic solar cells and OLEDs. The PAH101 dataset also contains band structures, exciton binding energies, and static dielectric constants. Notably, a few of the materials in the dataset have particularly low singlet exciton binding energies and particularly high dielectric constants. In addition to containing information on potentially useful materials, the dataset could be used to identify structure-property correlations.

In materials discovery workflows it is desirable to use models that are fast to evaluate for preliminary screening of a large number of candidates. Semi-local DFT has been used extensively for this purpose. However, such models must be sufficiently reliable to at least capture the correct trends. In the SI, we present statistical analysis across our dataset to examine whether selected DFT models are sufficiently predictive of *GW*+BSE quantities. The PAH101 dataset may similarly serve as a resource for researchers interested in comparing the results of other DFT and TDDFT models to *GW*+BSE.

To demonstrate how the PAH101 dataset can be reused to train ML models for other purposes than SF, we used SISSO to find predictive models for the *GW* fundamental band gap. The results are presented in the SI. In Ref. ^171^, we further used SISSO to train models to predict the optical gap, the triplet exciton energy, the singlet-triplet gap, and the singlet exciton binding energy. The dataset can be used in a similar manner to train ML models other than SISSO to predict any of the quantities included in the dataset. In addition, it can be used to supplement larger lower-fidelity datasets to train multi-fidelity models.

## Supplementary information

Supplementary Information

## Data Availability

• The *HAppend* code for adding missing hydrogen atoms to molecular crystal structures is available in the GitHub Repository HAppend (10.5281/zenodo.15093246), together with the pseudopotentials used in our calculations and scripts for making band structure and absorption plots.• Scripts for calculating the SISSO primary features and for processing SISSO results are available in the GitHub repository MLfeat_FHI-aims (10.5281/zenodo.15093306).• The BerkeleyGW code for performing *G**W*+BSE calculations^133^ is available at the BerkeleyGW website.• The FHI-aims code^128^, used to perform some relaxations and calculate DFT features, is available at the FHI-aims website. Version 18.06.07 was used here.• The Quantum ESPRESSO code^132^, used to calculate the mean-field wave functions for subsequent *G**W*+BSE calculations, is available at the Quantum ESPRESSO website.• The SISSO code^72^, used to perform sure independent screening and sparsifying operator model training, is available at the GitHub Repository SISSO. SISSO version 3.3 dated July 2023 was used here.• Scripts for preparing the input for SISSO, running the training and model evaluation, analyzing the SISSO output, and making Pareto plots and correlation plots between the SISSO model predictions and the true labels are provided in the GitHub repository SISSOonPAH (10.5281/zenodo.15093308).
