# Supplementary material for: PAH101: A GW+BSE Dataset of 101 Polycyclic Aromatic Hydrocarbon (PAH) Molecular Crystals
Source: Sci Data. 2025 Apr 23;12:679. doi: 10.1038/s41597-025-04959-0 (PMC12019249; doi:10.1038/s41597-025-04959-0)
Supplement: Supplementary file 1 — Supplementary Information [file 41597_2025_4959_MOESM1_ESM.pdf]

Supplementary Information: PAH101: A GW+BSE Dataset of 101 Polycyclic Aromatic Hydrocarbon (PAH) Molecular Crystals

Siyu Gao[a£,†] Xingyu Liu[b£,†] Yiqun Luo,[‡] Xiaopeng Wang,[¶] Kaiji Zhao,[†] Vincent Chang,[†] Bohdan Schatschneider,[§] and Noa Marom[*,†,‡,‖]

†*Department of Materials Science and Engineering, Carnegie Mellon University, Pittsburgh, PA, 15213*

‡*Department of Physics, Carnegie Mellon University, Pittsburgh, PA, 15213*

¶*School of Foundational Education, University of Health and Rehabilitation Sciences, Qingdao 266113, China*

§*Department of Chemistry and Biochemistry, California State Polytechnic University at Pomona, Pomona, CA, 91768, USA*

‖*Department of Chemistry, Carnegie Mellon University, Pittsburgh, PA, 15213*

E-mail: nmarom@andrew.cmu.edu

£ These authors contributed equally to this work.

Materials Discovery

The electronic and optical properties of most of the materials in the PAH101 set have not been thoroughly investigated experimentally. Some of the quantities calculated here, such as triplet excitation energies, are difficult to probe experimentally and require highly specialized techniques and facilities. Therefore, although the PAH101 set is relatively small, it is possible that some useful materials would be found in it. Here, we provide examples for some of the electronic and optical properties relevant for organic electronic devices that can be extracted from the dataset. The dataset can be searched for materials with a particular property or combination of properties. As demonstrated below, the dataset may provide insights on structure-property relations and expose gaps in our understanding of the properties of molecular crystals that call for further investigation.

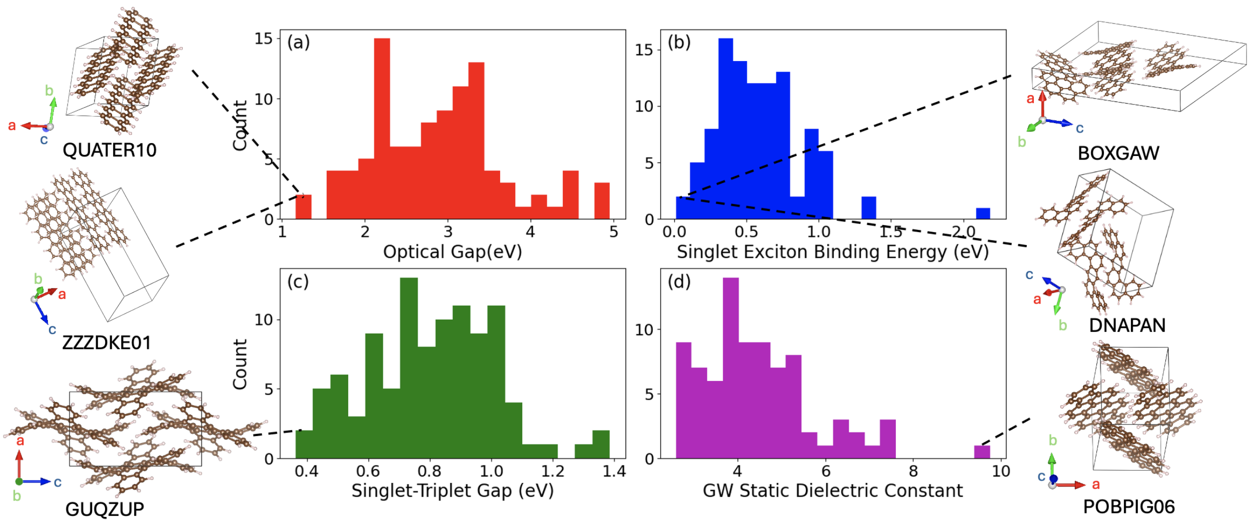

Supplementary Figure 1: Distributions of (a) the singlet exciton energies, which correspond to the optical gaps, (b) the singlet exciton binding energies, (c) the singlet-triplet gaps, and (d) the *GW* static dielectric constant across the PAH101 dataset. Some crystal structures are also shown.

One of the key properties for device applications is the optical gap, whose distribution in the dataset is shown in Supplementary Figure 1a. The PAH101 set contains materials with a wide range of optical gaps. Crystalline quaterrylene (QUATER10) and hexacene (ZZZDKE01) have the smallest optical gaps of 1.33 eV and 1.17 eV, respectively. Absorption

spectra for light polarized along the three crystal axes are also provided in the dataset, such that materials can be sought with broad absorption and/or absorption peaks in certain energy ranges.

The singlet exciton binding energy, whose distribution is shown in Figure 1b, corresponds to the difference between the *GW* fundamental gap and the optical gap. This is the energy required to split photogenerated excitons into free charge carriers in organic solar cells. In most organic materials the exciton binding energy is significant compared to inorganic materials because the dielectric screening of charges is not as strong. However, some materials in the PAH101 set have low exciton binding energies (in parentheses), including: anthra(2,1,9,8-hijkl)benzo(de)naphtho(2,1,8,7-stuv)pentacene (BOXGAW; 0.013 eV), dinaphtho(1,2-a:1',2'-h)anthracene (DNAPAN; 0.071 eV), tetrabenzo(de,no,st,c1d1)heptacene (TBZHCE; 0.130 eV), benzo[lm]chryseno[1,12,11,10-opqrab]perylene (YUNYAJ; 0.165 eV), and hexabenzo-(bc,ef,hi,kl,no,qr)coronene (HBZCOR; 0.169 eV). All of these compounds are characterized by very extended and/or elongated π systems, which likely lead to an already low molecular exciton binding energy (not calculated here), further reduced by dielectric screening in the solid form. Triplet exciton binding energies are also provided in the dataset. They are typically significantly higher than singlet exciton binding energies.

Another property of interest for device applications is the singlet-triplet gap, *i.e.*, the energy difference between the lowest singlet excited state and the lowest triplet excited state, both of which are included in the PAH101 dataset. The singlet-triplet gap is a key property for organic light emitting diodes (OLEDs). Most of the electrically generated excitons in OLEDs are triplet excitons, which cannot decay radiatively to the ground state. In thermally activated delayed fluorescence (TADF) chromophores, a small singlet-triplet gap enables reverse intersystem crossing (RISC) from the lowest triplet excited state to the lowest singlet excited state, which subsequently decays to the ground state, emitting a photon.[1–3] Figure 1c shows the distribution of singlet-triplet gaps in the PAH101 dataset. Small singlet-triplet gaps are rare among this class of materials. The materials with low-

est singlet-triplet gaps (in parentheses) are: trinaphtho[1,2,3,4-fgh:1',2',3',4'-pqr:1",2",3",4"-za_1_b_1_]trinaphthylene (GUQZUP; 0.36 eV), 9,18-diphenyltetrabenz(a,c,h,j)anthracene (FACPEE; 0.38 eV), acenaphtho[3,2,1,8-fghij]tetrabenzo[a,c,m,o]picene (VUFHUA; 0.435 eV), benzo(1,2,3-bc:4,5,6-b',c')dicoronene (YOFCUR; 0.44 eV), and 2-(naphthalen-2-yl)azulene (PUJQIV; 0.45 eV). Even the lowest singlet-triplet gaps in the PAH101 set would be considered marginal or too high for TADF. However, examining these materials may reveal new classes of chromophores that could be interesting for further investigation and fine-tuning by chemical modification. Charge transfer (CT) excitations between spatially separated HOMO and LUMO states are considered key to achieving small singlet-triplet gaps in TADF chromophores.[2,4] With the exception of PUJQIV, the materials with the smallest singlet-triplet gaps in the PAH101 set bear no resemblance to the donor-acceptor compounds typically used for TADF. Rather, they are large PAHs with extended π systems. FACPEE, VUFHUA, and YOFCUR have segments that could lead to CT-like intramolecular excitations. GUQZUP (shown in Figure 1c) can be described as a graphene flake with no obvious segments. The twisted conformation it adopts in the crystal structure may contribute to orbital localization and CT-like excitations. The effect of crystal packing and intermolecular vs. intramolecular CT excitations on singlet-triplet gaps is also not well-understood and should be further investigated in relation to TADF in crystalline materials.[5,6]

Figure 1d shows the distribution of the *GW* static dielectric constant in the PAH101 dataset. Several materials in the dataset have high *GW* static dielectric constants (in parentheses), including: diindeno[1,2,3-cd:1',2',3'-lm]perylene (POBPIG06; 9.75), benzo[lm]-chryseno[1,12,11,10-opqrab]perylene (YUNYAJ; 7.51), hexacene (ZZZDKE01; 7.41), indeno-(7,7a,1,2,3-lmno)-1,12-ethenochrysene (SURTAA; 7.33), and tetrabenzo[a,d,j,m]coronene (SETTES; 7.05). These are compounds with extended and/or elongated π systems, which are probably highly polarizable (the molecular polarizability is not calculated here). The crystal packing probably also contributes significantly to the dielectric screening. Most of the research on organic materials with high dielectric constants has been on polymers for

applications in bulk heterojunction organic solar cells (*e.g.*,[7]), which are very different from the materials in the PAH101 set. This calls for further investigation of the dielectric behavior of molecular crystals. We note that the full dielectric function, which contains information on the frequency dependence and anisotropy, is available in the dataset.

Correlations between DFT features and *GW*+BSE

Supplementary Figure 2 shows correlation plots between selected primary features calculated by DFT with the PBE functional and *GW*+BSE@PBE. We note that the DFT primary features used here were computed for locally optimized geometries as described in the Methods Section in the main text. In Panel (a) single-molecule and crystal DFT quantities are compared to the *GW*+BSE crystal optical gap. The fundamental gap of a molecule corresponds to the difference between the ionization potential (IP) and electron affinity (EA). The fundamental gap of a molecular crystal (calculated by *GW*) is typically significantly narrower than the single molecule fundamental gap because of screening and band dispersion in the crystal.[8] The optical gap of a molecular crystal is narrower than the fundamental gap because of the exciton binding energy.[9] The IP and EA calculated based on on DFT total energy differences are better estimates than the Kohn-Sham eigenvalues of the HOMO and LUMO. However, it has been shown that the molecular fundamental gaps obtained from PBE IP-EA have errors of 0.89 eV on average compared to reference data.[10] As expected, the molecular PBE IP-EA values significantly overestimate the *GW*+BSE optical gaps of the corresponding molecular crystal. Although there is correlation with the overall trend of the *GW*+BSE optical gaps, the spread of the PBE IP-EA values is too large to be considered as a reliable predictor.

It is well known that molecular HOMO-LUMO gaps and crystal band gaps are significantly underestimated by (semi-)local functionals such as PBE, owing to the self-interaction error (SIE).[11] For the PAH101 set, both the PBE single molecule HOMO-LUMO gap and

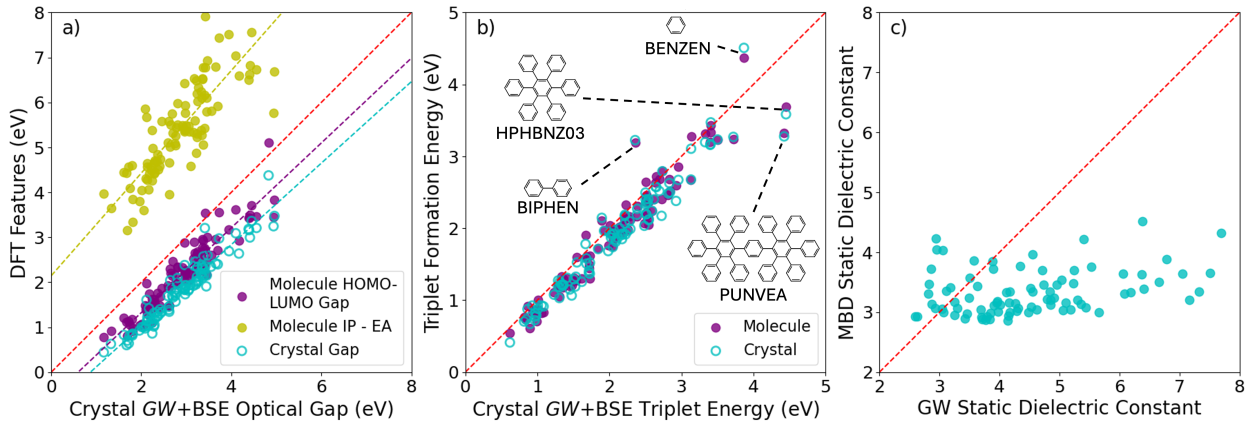

Supplementary Figure 2: Correlations between DFT and GW+BSE across the PAH101 set for selected properties: (a) DFT molecular IP-EA, molecular HOMO-LUMO gaps, and crystal band gaps compared to GW+BSE optical gaps. (b) DFT triplet formation energy of the molecule and crystal compared to the GW+BSE triplet exciton energy. Molecular structures of some outliers are also shown. (c) DFT dielectric constant calculated by using the MBD polarizability in the Clausius-Mossotti equation compared with the GW static dielectric constant.

crystal gap systematically underestimate but correlate well with the GW+PBE optical gaps. Based on this, these values may be sufficiently reliable for rough preliminary screening based on relative trends among materials. The single molecule PBE HOMO-LUMO gap is particularly attractive for this purpose because it is very fast to evaluate. Furthermore, there are large datasets of single molecule[12] and crystal PBE gap[13] that can be mined. We note, however, the effect of SIE is material-dependent.[14,15] Compounds whose HOMO and/or LUMO are highly localized may be affected more severely than PAHs, whose frontier molecular orbitals are typically delocalized over the aromatic system. Therefore, it would be prudent to reevaluate the reliability of DFT-PBE molecular and crystal gaps for more diverse data sets.

In Panel (b) the single molecule and crystal DFT triplet formation energies are compared to the GW+BSE triplet excitation energies. Overall, the single molecule and crystal DFT values are quite close to each other and to the GW+BSE triplet exciton energies, with MAEs of 0.20 eV and 0.23 eV, respectively and R^2 values of 0.89 and 0.86, respectively. The reasons for this agreement need to be investigated further (we are not aware of any benchmark stud-

ies of DFT triplet formation energies). The four most significant outliers, whose molecular structures are shown, are: biphenyl (BIPHEN), benzene (BENZEN), 2',2"',3',3"',5',5"',6',6"'-octaphenyl-p–quinquephenyl (PUNVEA), and hexaphenylbenzene (HPHBNZ03). These compounds are characterized by phenyl rings connected by single C-C bonds, whereas the majority of compounds in the PAH101 set are characterized by extended aromatic systems. Our results indicate that DFT triplet formation energies are fairly reliable as lower-cost descriptors for preliminary screening. However, based on the nature of the outliers, it would be prudent to validate these findings for more diverse materials.

Panel (c) shows a comparison between the static dielectric constant calculated by PBE+MBD and by GW. The GW value corresponds to the dielectric function value at 0 frequency and 0 wave-vector, $\epsilon(\omega = 0, q = 0)$. The DFT value is obtained by using the MBD polarizability in the Clausius-Mossotti relation, as described in Ref.[8] The comparison reveals that the DFT values are narrowly distributed around 3 and, in general, do not correlate with the GW values. For some materials the values obtained from PBE+MBD may fortuitously agree with experimental and/or GW values;[16] however, even with the self-consistent screening approach used in the MBD method,[17,18] DFT does not capture the many-body physics contained in the GW dielectric function. This demonstrates that it is important to consider larger sets of materials to assess the reliability of methods.

ML models for the *GW* fundamental gap

To demonstrate how the PAH101 dataset can be reused to train ML models for other purposes than SF, we use SISSO to find predictive models for the GW fundamental band gap, whose distribution in the PAH101 dataset is shown in Supplementary Figure3a. SISSO models were trained following the same procedure used in Ref.[19] The same primary features were used (also provided in the PAH101 dataset), with the exception of DF_s and DF_c, because the DFT estimate for the SF driving force is not a physically meaningful descriptor in relation

to the fundamental band gap. The same 10 structures as in Ref.[19] were withheld as an unseen test set and the remaining 91 structures were used for model training. Models were constructed with a maximum rung (the number of times primary features are combined) of 3 and a maximum dimension (Dim) of 4. Features were combined using the operator set $H = \{+, -, \times, \div, \exp, \log, ()^{-1}, ()^2, ()^3, \sqrt{}, \sqrt[3]{}, |\cdot|\}$. The maximum complexity, i.e., the maximum number of operators in one combined feature, was set to 10. A total of 5×10^2, 4×10^5, and 6×10^{10} features were generated by SISSO with a rung of 1, 2, and 3, respectively.

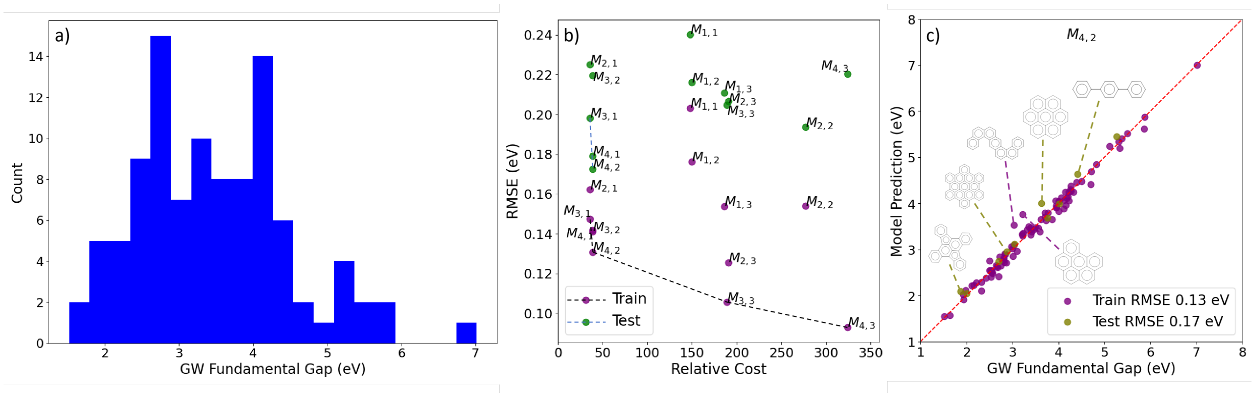

Supplementary Figure 3: Performance of SISSO-generated models for predicting the *GW* fundamental band gaps of molecular crystals: (a) Distribution of the *GW* fundamental band gap values in the PAH101 dataset. (b) Pareto chart of the accuracy vs. the computational cost of SISSO-generated models. The “train" accuracy corresponds to the RMSE obtained for the LCV validation set during training and the “test" accuracy corresponds to the withheld set of 10 materials not included in the training. The dashed lines indicate the Pareto front. (c) Model prediction as a function of the *GW* fundamental band gap for $M_{4,2}$. Molecular structures of some of the outliers are also shown.

After feature generation, SISSO performs linear regression to yield the model prediction, where each model is the scalar product of the SISSO-generated feature with a vector of fitted coefficients. Then, the models are ranked according to their prediction performance. Sure independence screening (SIS) is used to select optimal subspaces from the huge feature space. The number of features saved after SIS was set to 20. SISSO then uses ℓ_0-norm minimization as a sparsifying operator (SO) to determine the sparse solution for each such subspace. For each combination of dimension and rung, 40 rounds of leave-10-out cross validation (LCV) were performed. In each round, 10 data points (out of the 91 points used for model training)

were randomly selected and held out as an unseen validation set. The model with the lowest RMSE for the validation set was selected in each round. Finally, the model with the lowest root mean square error (RMSE) for the combined LCV training and validation data was selected out of the 40 models. This model is denoted as $M_{\text{Dim,Rung}}$. A full account of the SISSO models is provided below. Interestingly, SISSO does not produce any models that can predict the crystal fundamental gap based only on single molecule features.

Supplementary Table 1: The cost of SISSO primary features evaluated as a multiple of the cost of a PBE calculation for a single molecule in the ground state, averaged over 10 representative materials.

DFT Feature	Relative Cost
Gap^S	1
E_T^S	3
IP^S	2
EA^S	3
$PolarTensor^S$	2
Gap^C	33
VB_{disp}^C	33
CB_{disp}^C	33
H_{ab}	93
E_T^C	148
ϵ^C	42
$MolWt^S$	0
ρ^C	0
$AtomNum^C$	0

The computational cost of SISSO-generated models varies depending on the number and type of primary features they contain. The cost of each model was evaluated by summing over the costs of all the primary features included in it. The cost of features that appear in the model more than once was counted only once. The computer time required to calculate the single molecule PBE gap, Gap^S, was assigned a value of 1 cost unit and the cost of other features is tabulated in Supplementary Table 1. The cost of all the primary features has been updated from the values given in Ref.[19] to account for new developments in the latest version of FHI-aims. In particular, the MBD calculation has become significantly more efficient than in older versions of the code. The cost was averaged over the 10 structures in the validation

set, rather than picking one system of average size, as in Ref.[19] Supplementary Figure 3b shows a Pareto chart of the accuracy vs. the computational cost of the SISSO models considered here. The "train" RMSE is calculated for the training set of 91 structures. The "test" RMSE is calculated for the 10 withheld materials, which were excluded from the LCV. The best balance of cost and accuracy is provided by the $M_{4,2}$ model, which yields an RMSE of 0.13 eV for the training set and 0.17 eV for the unseen validation set. Supplementary Figure 3c shows a parity plot of the model prediction as a function of the *GW* reference values. Overall, the model performs well with few outliers. The $M_{3,3}$ and $M_{4,3}$ models, whose computational cost is considerably higher, have a better accuracy for the training set. However, their RMSE increases significantly for the unseen test set, which is indicative of over-fitting.

SISSO-generated models

$$M_{1,1} = 0.90 \times E_T^C/\rho^C + 1.32$$

$$M_{2,1} = 0.61 \times (E_T^S + Gap^C) - 1.6 \times (\rho^C)^3 + 1.99$$

$$M_{3,1} = 0.59 \times (E_T^S + Gap^C) + 0.00031 \times Gap^C \times AtomNum^C - 1.4 \times e^{\rho^C} + 4.23$$

$$M_{4,1} = 0.54 \times (E_T^S + Gap^C) + 0.00034 \times Gap^C \times AtomNum^C - 0.030 \times (EA^S)^3 - 2.6 \times \ln(\rho^C) + 0.79$$

$$M_{1,2} = 2.6 \times \frac{E_T^C}{\rho^C \sqrt{IP^S}} + 1.09$$

$$M_{2,2} = 6.9 \times \frac{E_T^C + H_{ab}}{IP^S \times \rho^C} - 20 \times \frac{Gap^C - Gap^S}{MolWt^S \times CB_{disp}^C} + 0.81$$

$$M_{3,2} = 3.8 \times \frac{Gap^C + Gap^S}{IP^S \times \rho^C} + 0.036 \times \frac{Gap^S - IP^S}{CB_{disp}^C - IP^S} + 52 \times \frac{(CB_{disp}^C)^3}{EA^S \times AtomNum^C} - 0.35$$

$$M_{4,2} = 0.90 \times \frac{E_T^S \times Gap^C}{Gap^S \times \rho^C} - 0.063 \times \frac{ln(CB_{disp}^C) \times AtomNum^C}{MolWt^S} + 197 \times \frac{(CB_{disp}^C)^3}{EA^S \times MolWt^S} + 0.035 \times \frac{EA^S}{Gap^S \times \ln(\rho^C)} + 1.67$$

$$M_{1,3} = 4.1 \times \frac{(E_T^S + IP^S) \times \sqrt{E_T^C}}{e^{\rho^C} \times (IP^S + CB_{disp}^C)} + 0.32$$

$$M_{2,3} = 4.1 \times \left[\ln(IP^S)/e^{\rho^C} + \frac{E_T^S + Gap^C}{IP^S + CB_{disp}^C} \right] - 3.3 \times \frac{EA^S \times CB_{disp}^C \times Gap^S}{PolarTensor^S \left| E_T^C + CB_{disp}^C - Gap^S + VB_{disp}^C \right|} - 2.27$$

$$M_{3,3} = 4.2 \times \left[\ln(IP^S)/e^{\rho^C} + \frac{E_T^S + Gap^C}{IP^S + CB_{disp}^C} \right] - 0.043 \frac{EA^S \times E_T^C \times (CB_{disp}^C)^2}{\left| E_T^C + CB_{disp}^C - Gap^S + VB_{disp}^C \right|} + 158 \times \frac{CB_{disp}^C \times (CB_{disp}^C - VB_{disp}^C)}{MolWt^S \times (Gap^C - |EA^S - E_T^C|)} - 2.28$$

$$M_{4,3} = 4.1 \times \left[\ln(IP^S)/e^{\rho^C} + \frac{E_T^S + Gap^C}{IP^S + CB_{disp}^C} \right] - 0.043 \times \frac{EA^S \times Gap^S \times (CB_{disp}^C)^2}{\left| E_T^C + CB_{disp}^C - Gap^S + VB_{disp}^C \right|} + 183 \times \frac{(CB_{disp}^C - VB_{disp}^C)}{MolWt^S \times \epsilon^C \times (Gap^C - H_{ab} - |EA^S - E_T^C|)} - 0.00028 \times \frac{(Gap^C)^2}{|H_{ab} - VB_{disp}^C| \times \left| Gap^S + CB_{disp}^C - |EA^S - VB_{disp}^C| \right|} - 2.17$$

Supplementary References

(1) Parker, C.; Hatchard, C. Triplet-singlet emission in fluid solutions. Phosphorescence of eosin. *Transactions of the Faraday Society* **1961**, *57*, 1894–1904.

(2) Endo, A.; Sato, K.; Yoshimura, K.; Kai, T.; Kawada, A.; Miyazaki, H.; Adachi, C. Efficient up-conversion of triplet excitons into a singlet state and its application for organic light emitting diodes. *Applied Physics Letters* **2011**, *98*.

(3) Yang, Z.; Mao, Z.; Xie, Z.; Zhang, Y.; Liu, S.; Zhao, J.; Xu, J.; Chi, Z.; Aldred, M. P. Recent advances in organic thermally activated delayed fluorescence materials. *Chemical Society Reviews* **2017**, *46*, 915–1016.

(4) Chen, X.-K.; Kim, D.; Brédas, J.-L. Thermally activated delayed fluorescence (TADF) path toward efficient electroluminescence in purely organic materials: molecular level insight. *Accounts of Chemical Research* **2018**, *51*, 2215–2224.

(5) Cai, X.; Qiao, Z.; Li, M.; Wu, X.; He, Y.; Jiang, X.; Cao, Y.; Su, S.-J. Purely organic crystals exhibit bright thermally activated delayed fluorescence. *Angewandte Chemie International Edition* **2019**, *58*, 13522–13531.

(6) Zhan, L.; Chen, Z.; Gong, S.; Xiang, Y.; Ni, F.; Zeng, X.; Xie, G.; Yang, C. A simple organic molecule realizing simultaneous TADF, RTP, AIE, and mechanoluminescence: understanding the mechanism behind the multifunctional emitter. *Angewandte Chemie* **2019**, *131*, 17815–17819.

(7) Brebels, J.; Manca, J. V.; Lutsen, L.; Vanderzande, D.; Maes, W. High dielectric constant conjugated materials for organic photovoltaics. *J. Mater. Chem. A* **2017**, *5*, 24037–24050.

(8) Wang, X.; Liu, X.; Tom, R.; Cook, C.; Schatschneider, B.; Marom, N. Phenylated acene derivatives as candidates for intermolecular singlet fission. *The Journal of Physical Chemistry C* **2019**, *123*, 5890–5899.

(9) Sharifzadeh, S.; Biller, A.; Kronik, L.; Neaton, J. B. Quasiparticle and optical spectroscopy of the organic semiconductors pentacene and PTCDA from first principles. *Phys. Rev. B* **2012**, *85*, 125307.

(10) Gallandi, L.; Marom, N.; Rinke, P.; Körzdörfer, T. Accurate Ionization Potentials and Electron Affinities of Acceptor Molecules II: Non-Empirically Tuned Long-Range Corrected Hybrid Functionals. *Journal of Chemical Theory and Computation* **2016**, *12*, 605–614.

(11) Golze, D.; Dvorak, M.; Rinke, P. The GW compendium: A practical guide to theoretical photoemission spectroscopy. *Frontiers in chemistry* **2019**, *7*, 377.

(12) Stuke, A.; Kunkel, C.; Golze, D.; Todorović, M.; Margraf, J. T.; Reuter, K.; Rinke, P.; Oberhofer, H. Atomic structures and orbital energies of 61,489 crystal-forming organic molecules. *Scientific data* **2020**, *7*, 58.

(13) Olsthoorn, B.; Geilhufe, R. M.; Borysov, S. S.; Balatsky, A. V. Band Gap Prediction for Large Organic Crystal Structures with Machine Learning. *Advanced Quantum Technologies* **2019**, *2*, 1900023.

(14) Knight, J. W.; Wang, X.; Gallandi, L.; Dolgounitcheva, O.; Ren, X.; Ortiz, J. V.; Rinke, P.; Korzdorfer, T.; Marom, N. Accurate ionization potentials and electron affinities of acceptor molecules III: a benchmark of GW methods. *Journal of chemical theory and computation* **2016**, *12*, 615–626.

(15) Marom, N.; Caruso, F.; Ren, X.; Hofmann, O. T.; Körzdörfer, T.; Chelikowsky, J. R.; Rubio, A.; Scheffler, M.; Rinke, P. Benchmark of G W methods for azabenzenes. *Physical Review B* **2012**, *86*, 245127.

(16) Schatschneider, B.; Liang, J.-J.; Reilly, A. M.; Marom, N.; Zhang, G.-X.; Tkatchenko, A. Electrodynamic response and stability of molecular crystals. *Phys. Rev. B* **2013**, *87*, 060104.

(17) Ambrosetti, A.; Reilly, A. M.; DiStasio, R. A.; Tkatchenko, A. Long-range correlation energy calculated from coupled atomic response functions. *The Journal of chemical physics* **2014**, *140*.

(18) Tkatchenko, A.; DiStasio, R. A.; Car, R.; Scheffler, M. Accurate and Efficient Method for Many-Body van der Waals Interactions. *Phys. Rev. Lett.* **2012**, *108*, 236402.

(19) Liu, X.; Wang, X.; Gao, S.; Chang, V.; Tom, R.; Yu, M.; Ghiringhelli, L. M.; Marom, N. Finding predictive models for singlet fission by machine learning. *npj Computational Materials* **2022**, *8*, 70.
